# Isolation, characterization, and bioremediation potential of oil-degrading bacteria from contaminated soils in Shiraz and Bandar Abbas, Iran

**DOI:** 10.1038/s41598-025-26242-3

**Published:** 2025-11-26

**Authors:** Sara Shokranian, Morteza Yousefzadi, Narges Amrollahi Biuki, Mohammad Yaghoubi-Avini, Majedeh TaheriAkerdi

**Affiliations:** 1 Environmental and Industrial Biotechnology, Department of Marine Biology, Faculty of Marine Sciences and Technology, University of Hormozgan, Bandar Abbas, Iran; 2 Department of Biology, Faculty of Sciences, University of Qom, Qom, Iran; 3 Department of Marine Biology, Faculty of Marine Sciences and Technology, University of Hormozgan, Bandar Abbas, Iran; 4 Department of Microbiology and Microbial Biotechnology, Faculty of Life Sciences and Biotechnology, Shahid Beheshti University, Tehran, Iran; 5 Department of Biotechnology, Faculty of Science and Strategic Technologies, University of Qom, Qom, Iran

**Keywords:** Biodegradation, Biosurfactant, Antagonism, *Acinetobacter junii*, *Lysinibacillus boronitolerans*, Biotechnology, Environmental sciences, Microbiology

## Abstract

Petroleum hydrocarbons are among the most persistent environmental pollutants, posing serious risks to soil and aquatic ecosystems. Bioremediation using indigenous hydrocarbon-degrading bacteria provides a cost-effective and environmentally sustainable alternative to physical and chemical treatments. In this study, oil-degrading bacteria were isolated from contaminated soils collected near oil refineries in Shiraz and Bandar Abbas, Iran, and screened for their biodegradation potential. A total of twenty-four bacterial isolates were obtained, among which four showed the highest growth rates and crude-oil degradation efficiency. These isolates were further evaluated for biosurfactant production using hemolysis, drop-collapse, oil displacement, emulsification (E24), and bacterial adherence to hydrocarbon (BATH) assays, as well as for hydrocarbon removal by spectrophotometry and gas chromatography with flame ionization detection (GC-FID). In biosurfactant assays, SHA showed significantly higher E24 and BATH values than the other isolates (*P* < 0.05; Table 3). By spectrophotometry and GC-FID, SHA and SWOC achieved the highest crude-oil removal after seven days (84.2% and 85.8%, respectively), and GC-FID confirmed degradation across C13–C32 n-alkanes.Molecular identification based on 16S rRNA sequencing revealed that isolate SHA belonged to *Acinetobacter junii* (Gram-negative) and isolate SWOC to *Lysinibacillus boronitolerans* (Gram-positive). Interestingly, co-cultivation of the two isolates resulted in reduced degradation efficiency (55%), indicating an antagonistic interaction between them. These findings suggest that both isolates possess strong individual potential for the bioremediation of oil-contaminated soils, and further studies are warranted to investigate the mechanistic basis of antagonism and to validate their performance under field conditions.

## Introduction

Crude oil is a complex mixture of hydrocarbons with short and long chains, which are divided into four main categories: saturated hydrocarbons, polycyclic aromatic hydrocarbons (PAHs), asphaltenes, and resins. Crude oil that contains saturated and aromatic hydrocarbons is called light oil, and crude oil that contains resins and asphaltenes is called heavy oil^1–3^.

Oil pollution is an inevitable result of industrial activities, and it can have a devastating impact on the environment. It can occur through accidents involving oil tankers, leaks from pipelines, and offshore platforms^4,5^. From 1970 to 2014, an estimated 5.74 million tons of oil entered the oceans as a result of oil tanker accidents. Petroleum hydrocarbons are among the most common pollutants, and they can cause significant damage to marine environments, soil, and the health of living organisms^6–8^.

In recent years, due to human activities such as maritime transportation, various pollutants have entered the open waters, which have endangered the lives of many organisms^1^. Based on research, the annual input of crude oil to the world’s waters is about 1.3 million tons. Since the solubility of petroleum hydrocarbons in water is very low, they often remain floating on the water and form a thin layer on the surface of the water, which prevents the passage of enough oxygen to the underlying layers of water and endangers the lives of marine animals^2,9^. Moreover, sometimes heavier particles penetrate into the underlying layers of water and settle in marine sediments. When crude oil enters the marine environment, it enters the food chain of marine animals and remains in that environment for years, leaving harmful effects on biological systems^10^.

Oil pollution can not only change aquatic and terrestrial environments, but also can have harmful effects on the health of humans and other living things, such as carcinogenicity, mutagenicity, and lifestyle change. According to a report by ATSDR, (Agency for Toxic Substances and Disease Registry.) PAHs are the most toxic compounds in crude oil, which are highly carcinogenic^6,11^.

In general, three methods are used to remove environmental oil pollution: physical, chemical, and biological. Physical methods require expensive technologies and sometimes lead to incomplete decomposition of pollutants. On the other hand, chemical methods are also very expensive and can cause secondary pollution^12^. Bioremediation is one of the safe and affordable biological methods that can be used in hydrocarbon-contaminated areas^2^.

Bioremediation is an environmentally friendly method that can be used to some extent to quickly remove environmental pollutants from water and soil^6^. Bioremediation is a process in which chemical compounds are converted into energy, cellular mass, and biological waste by living organisms, especially microorganisms. In the case of petroleum hydrocarbons, these biological wastes are mainly CO2, water, and methane^13,14^.

The history of the discovery of the first hydrocarbon-degrading bacteria dates back to about a century ago. A wide range of microorganisms have the ability to decompose petroleum hydrocarbons. According to reports, over 360 genera of bacteria and fungi are involved in the decomposition of petroleum hydrocarbons^15^. In recent years, scientists have identified 14 genera of algae, 103 genera of fungi, 79 genera of bacteria, and nine genera of cyanobacteria that have the ability to decompose and convert hydrocarbons^2^. Among these, algae and protozoa play a lesser role in the biodegradation of hydrocarbons^7^.

To date, more than 79 genera of bacteria that are capable of decomposing hydrocarbons have been identified^16^. The use of aerobic bacteria is a more affordable and effective method for biodegrading petroleum pollutants than anaerobic bacteria. However, anaerobic bacteria have limitations compared to aerobic bacteria, such as being slower, less efficient, and having a more limited range of metabolic pathways^17^. As of 2015, more than 5 genera of anaerobic bacteria have been reported among 79 genera of hydrocarbon-degrading bacteria^9^. The most well-known genera of hydrocarbon-degrading bacteria include *Halomonas*, *Alcanivorax*, *Marinobacter*, *Dietzia*, *Bacillus*, *Oleiphilus*, *Oleispira*, *Geobacillus*, *Pseudomonas*, *Alcaligenes*, *Micrococcus*, *Methylomonas*, *Achromobacter*, *Acinetobacter*, *Arthrobacter*, *Nocardia*, *Flavobacterium*, *Corynebacterium*, *Methylobacterium*, and *Rhodococcus*^7,13^.

This research aims to assess aerobic oil-degrading bacteria isolated from contaminated soil which is effective for petroleum hydrocarbon bioremediation.

## Materials and methods

### Sampling

Samples were collected from 10 cm deep soil of oil-contaminated areas near two oil refineries in Bandar Abbas and Shiraz (Iran). The soil samples were transported on ice in sterile glass containers to the laboratory of the University of Hormozgan for further analysis. In addition, three liters of crude oil were provided by Bandar Abbas Oil Refinery for more studies.

### Isolation and selection of crude-oil degrading bacteria

For the purification and isolation of oil-eating bacteria, the samples were inoculated into the Bushnell Hass Mineral Salt (BHMS) culture medium at a ratio of 1% (v/v), and crude oil was added to the culture medium at a ratio of 1% (v/v) as the only source of carbon. The samples were incubated for 72 h at 30°C on a rotary shaker (WiseCube WIS-20, Germany) (160 rpm, INFORS AG). After this period, oil-consuming strains were separated by the serial dilution method. For this purpose, serial dilutions of 10^–1^ to 10^–5^ were prepared in a nutrient broth medium, and 100 µl of each dilution was cultured on a Nutrient Agar (NA) plate by spread plate technique. The plates were incubated for 72 h at 30°C. Then, the morphology of the colonies formed on the plates containing 30 to 300 colonies was examined^18^.

### Screening of selected oil-eating strains

The objective was to identify superior isolates, growth rates, and assess their oil consumption efficiency. For the oil consumption test, Bandar Abbas isolates were initially cultured in Mueller-Hinton broth (MHB) medium at 30°C overnight, and their absorbance was measured, maintaining it between 0.08 and 0.13 according to McFarland standards.

Subsequently, Erlenmeyer flasks containing BHMS culture medium, 1% crude oil, and 1% of each isolate were placed in a rotary incubator (Lovibond TC135, Germany) at 30°C for two weeks; Flasks without bacteria served as controls. Bacterial growth absorbance at 600 nm was measured four times during this period. This process was then repeated for Shiraz isolates, with a shorter 7-day test to emphasize bacterial speed of oil consumption.

The most effective isolates were selected and subcultured into the BHMS medium with 1% crude oil for seven days at 30°C and 160 rpm. The growth was monitored at days zero, three, and seven after inoculation.^2^.

### Biosurfactant production assay

Five methods were used for the detection of biosurfactant production as follows^19,20^:

### Blood hemolysis test

The selected strains were cultured on the blood agar (5% sheep blood). The plates were kept in the incubator at 30°C for 48–72 h. The development of clear zone (hemolysis) around the bacteria indicates the possibility of biosurfactant production. *Staphylococcus aureus* was used as a positive control^19,21^.

#### Drop-collapse test

Ten microliters of crude oil was poured on the slide and then cell-free culture supernatants were dropped on the oil. After 1 min, a conclusion was made based on the shape of the drop on the oil. the flattening of the droplets on the oil, as a result of the reduction of surface tension, was a testimony to the production of biosurfactants, otherwise the drop will be completely round. Distilled water and BHMS solution were used as negative control.

#### Oil displacement test

20 ml of distilled water was poured into a plate, and 20 µl of oil was added to its surface to form a thin, integrated layer on its surface. Then, 10 µl of cell-free culture supernatants were pipettedsuspension into the center of the oil, and the diameter of the clear halo was measured. In this test, water and BHMS medium were negative controls.

#### Emulsification activity (E24)

2 mL of each cell-free supernatant in the logarithmic phase of growth was added to a test tube containing 3 mL of kerosene and was shaken at high speed for 3 min^22^. The tubes were incubated at room temperature for 24 h, and after this time, the emulsification activity was calculated using the following formula:

$$\begin{aligned} \% {\text{Emulsification }} &= {\text{ (Height of emulsion zone/Total height of liquid) }}\\ &\quad \times 100 \\ \end{aligned}$$

#### Bacterial adherence to hydrocarbons (BATH)

A fresh culture of bacteria in MHB was centrifuged (Fixette II, Germany) at 10,000 rpm for 15 min. The pellets were resuspended in phosphate saline buffer, and the turbidity of all samples was adjusted to between 0.4 and 0.6 as the initial optical density (OD). 1 mL of xylene was added to this suspension and vortexed (Reax top—Heidolph, Germany) for 2 min. Then, the samples were left at room temperature for 45 min to separate the aqueous and organic phases. The turbidity of the aqueous phase was read at a wavelength of 600 nm and was recorded as the secondary OD (A2). The degree of hydrophobicity of the cell surface was calculated by the following formula:

$$\% {\text{Hydrophobicity }} = \, \left( {\left( {{\text{A2 }} - {\text{ A1}}} \right) \, /{\text{ A1}}} \right) \, \times { 1}00$$

### Determination of crude oil removal by selected bacteria

This step was performed by spectrophotometric tests and GC-FID (Gas chromatography—flame ionization detector.) assays^7,18^.

#### Spectrophotometric method

The bacteria were inoculated into 20 ml of BHMS medium containing 1% crude oil. After a 7-day incubation period, the growth of the strains was first evaluated by the spectrophotometer (Cecil BioAuest) at a wavelength of 600 nm. Then, 10 mL of dichloromethane (DCM) was added to the flasks, well-mixed, and transferred to a separatory funnel. A volume of 1 mL of the organic phase was combined with 5 mL of DCM, and the turbidity was read at 420 nm. The percentage of crude oil removal was calculated using the following formula:

$$\begin{aligned} & {\text{Percentage of crude}} - {\text{oil removal }}\left( \% \right) \, \\ & \quad = \, \left( {{\mathrm{A}}\_{\text{blank }} - {\text{ A}}\_{\mathrm{sample}}} \right) /{\text{ A}}\_{\text{blank }} \, \\ & \quad \times { 1}00{\text{ at 42}}0{\text{ nm}};{\text{ blanks contained BHMS }} \\ & \quad \quad + { 1}\% {\text{ crude oil without cells and were processed identically}}. \\ \end{aligned}$$

#### GC-FID method

The organic phase of the previous method was analyzed by a Varian CP-3800 instrument fitted by a CP Sil-5 column. Helium was used as a carrier gas. The initial temperature was 40 °C for 3 min and reached 280 °C by 10°/m. The injection port temperature was 280 °C, and the detector temperature was 300 °C. The split ratio was also 20.

The peak areas were compared with the standard, and the degradation percentage for each isolate was calculated.

Peak areas were compared with the corresponding peaks in the abiotic control. Where specific isomeric assignments were uncertain, peaks were reported as isomeric Cxx entries.

### Investigation of the effect of bacterial consortiums

The suspensions of the two selected strains, SWOC and SHA, were incubated for seven days in a BHMS culture medium containing 1% crude oil at 30°C in a shaking incubator at 160 rpm. Then, the oil removal percentage was determined by spectrophotometry, as mentioned above. Additionally, biosurfactant production measurements were repeated for the mixed bacteria to investigate its effect on biosurfactant production^23^.

### Identification of isolates

#### Biochemical and Molecular Identification

Biochemical identification, which included Gram staining, was studied using the^24^ method and then performed using ready-made kits purchased from Labtron Company, according to the instructions of the company.

To phylogenetically characterize the isolated strains, their *16S rRNA* genes were amplified using the boiling method to extract total DNA. The 9F (5′-AGAGTTTGATCCTGGCTCAG-3′) and 1541R (5′-AAGGAGGTGATCCAGCCGCA-3′) primers were used to amplify the *16S rRNA* loci of the bacteria. After obtaining the PCR (Bio Rad T100, Singapore) product, horizontal electrophoresis in a 1% agarose gel was performed to verify the success of the PCR reaction. The samples were sent to the GeneFanavaran company for Sanger sequencing. The obtained sequences were revised using the Chromas software. Finally, they were blasted in the NCBI database, and their similarity percentage was studied^18,25^.

#### Phylogenetic tree building

To build the phylogenetic tree, the sequences of the selected bacterial genes were compared to the sequences of the other similar bacteria in the GenBank. The sequences were aligned to eight 16s rRNA genes of near genera using the Clustal W option of the MEGAX software. The phylogenetic tree was drawn using the Kimura two-parameter model of the Neighbor-joining method and bootstrapped by 1000 replicates^26,27^.

### Statistical analysis

Data were analyzed using one-way ANOVA followed by Duncan’s multiple range test for mean separation at α = 0.05 (*P* < 0.05 considered significant). Results are reported as mean ± SD over biological replicates (n = 3). Analyses were performed in SPSS 20; figures were prepared in Excel 2016.

## Results

### Isolation and selection of crude-oil degrading bacteria

A total of 11 colonies with different morphologies were isolated from the Bandar Abbas samples and 13 colonies with different morphologies were isolated from the Shiraz samples. Four bacterial strains with the highest growth rates on crude oil were selected for further study (Tables 1 and 2). Two strains (LWOE and SWOC) originated from Bandar Abbas, and the others (Sludge C and SHA) originated from Shiraz.

**Table 1:** Growth absorption (OD) of Bandar Abbas isolates at 600 nm during 14 days († Isolate selected for in-depth analyses)

Isolation	Day zero	Day four	Day nine	Day fourteen
LWOA	0.074	0.145	0.053	0
LWOB	0.575	0.130	1.242	1.348
LWOC	0.357	0	0	0
LWOD	0.81	0.796	0.814	0.920
LWOE	0.626	0.193	1.472	1.600
L2OA	0.665	0.490	0.575	0.535
L2OB	0.689	0.662	0.682	0.323
L2OC	0.780	0.937	0.960	0.580
SWOA	0.074	0.086	0	0
SWOB	0.540	0.196	0.115	0.012
**†SWOC**	0.490	0.742	1.576	1.884

**Table 2:** Growth absorption (OD) of Shiraz isolates at 600 nm during seven days.

Isolation	Day zero	Day four	Day seven
Sludge A	0.07	0	0
Sludge B	0.127	0.183	0.448
Sludge C	0.658	1.038	1.194
Sludge D	0.053	0.162	0.087
Sludge E	0.066	0.035	0
SLA	0.051	0	0
SLB	0.057	0.105	0
SLC	0.116	0.307	0.667
SLD	0.097	0.099	0.098
**†SHA**	0.574	1	0.918
SHB	0.115	0.154	0.011
SHC	0.155	0.367	0.368
SHD	0.046	0.087	0

SHA and SWOC were selected for in-depth analyses (biosurfactant assays, spectrophotometry, GC-FID, and co-culture test).

### The growth curve of selected isolates

**Fig. 1:**
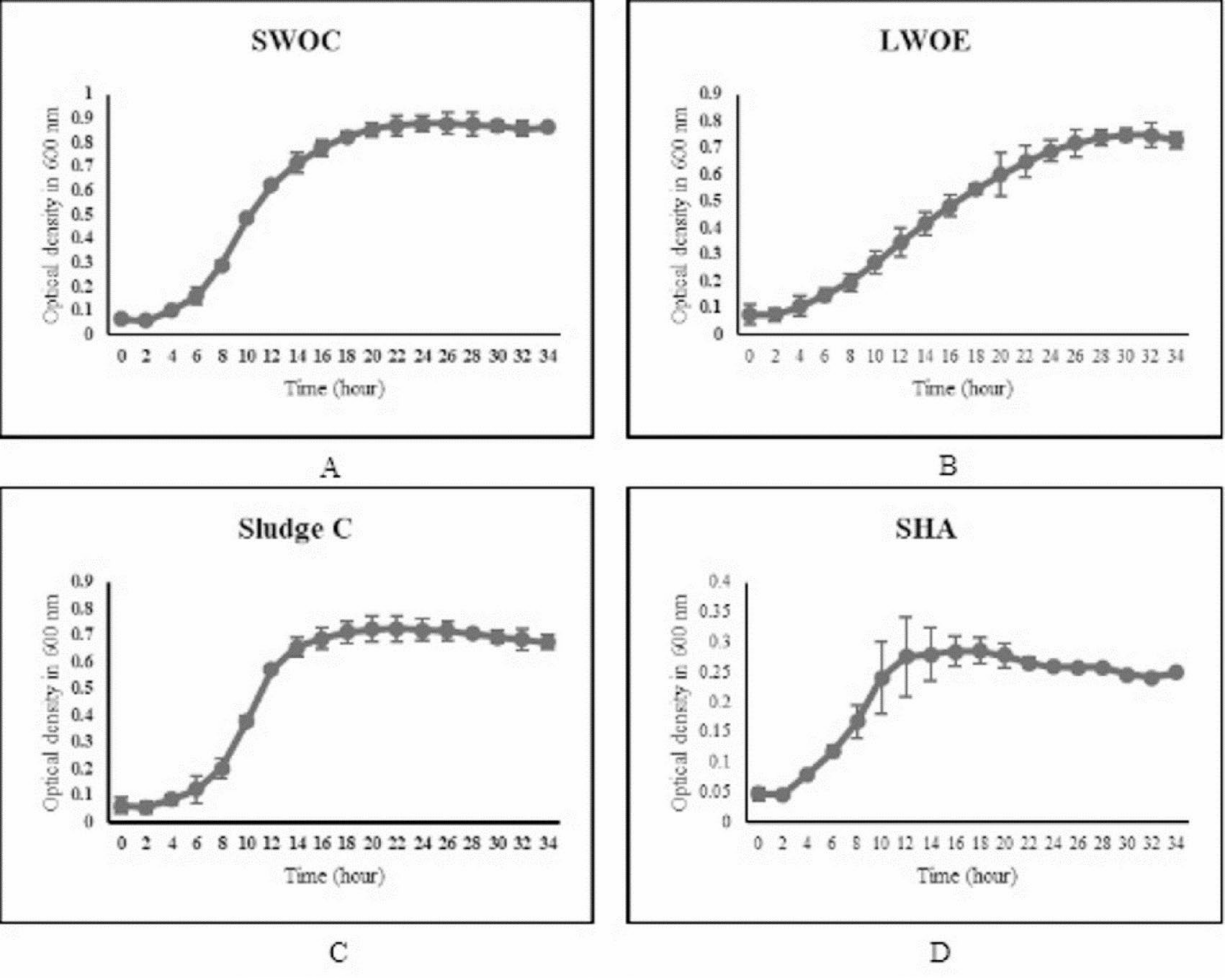
Growth curves of the SWOC, SludgeC, LWOE, and SHA isolates over a 36-h period, shown in graphs A, B, C, and D, respectively.

The logarithmic phase of all four selected isolates was studied (Fig. 1). It was observed that all of the isolates entered the logarithmic phase after 4 h, but the isolate LWOE had the longest logarithmic phase, which lasted for 24 h. The other three isolates remained in the lag phase for 12 h. The assay did not show any death phase within the 36 h of the study.

### Biosurfactant production assay results

#### Blood hemolysis test

**Fig. 2:**
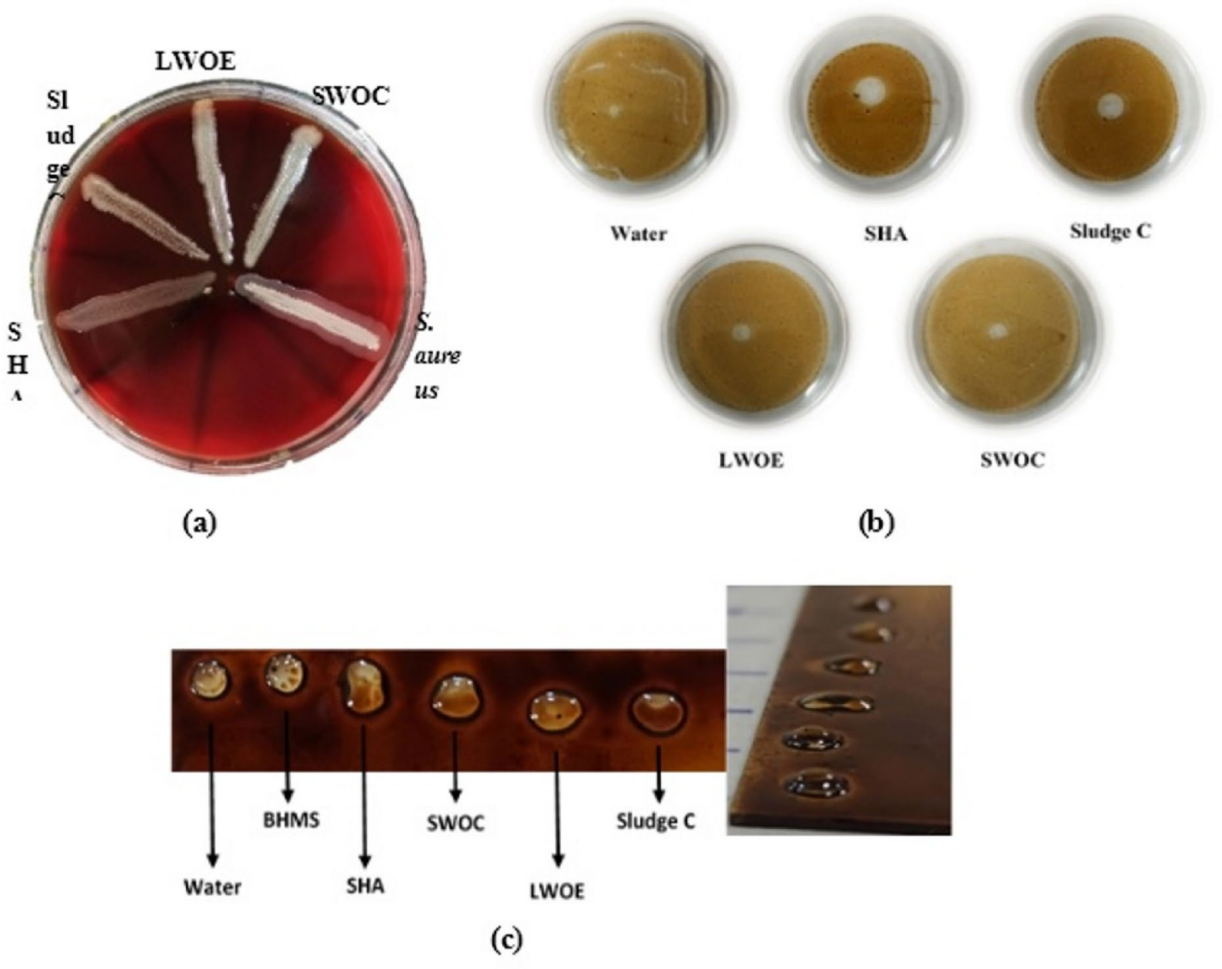
Results of biosurfactant production. Hemolysis of isolates in blood agar medium. SWOC, Sludge C, SHA, and LWOE isolates showed good hemolysis, but no hemolysis was observed in the L2OA and L2OB isolates. *S. aureus* was used as a control in this step (**a**). The displaced oil diameter formed by each isolate in the oil spreading assay. Distilled water was used as a control. The amount of oil spreading did not change appreciably in distilled water (**b**). Results of the drop-collapse assay. The more spherical and round the droplets, the less biosurfactant is present or produced, and the flatter and wider the droplets, the more biosurfactant is produced. Distilled water and BHMS medium were used as negative controls (**c**).

All four top isolates have hemolysis activity, which were consistent with their ability to produce biosurfactants (Fig. 2a).

#### Oil displacement measurement

The isolate SHA had the largest displaced circle among the other isolates and probably produced more biosurfactants. Distilled water was used as a control. In this test, all isolates formed a clear area on the oil surface, the highest amount of biosurfactant produced by SHA having a halo diameter of 0.9 cm (Fig. 2b). A comparison of oil spreading in each isolate is shown in Table 3.

**Table 3:** Summary of biosurfactant test results for SWOC, LWOE, SHA and Sludge C isolates.

Sample	SWOC	LWOE	Sludge C	SHA
Blood hemolysis	+	+	+	+
Oil displacement	0.33 ± 0.05^b^	0.4 ± 0.1^b^	0.83 ± 0.05^a^	0.93 ± 0.05^a^
Drop collaps	+ +	+ +	+	+ + +
E24	15.71 ± 1.43^c^	11.07 ± 2.17^d^	58.95 ± 1.57^b^	66.45 ± 0.66^a^
BATH	73.66 ± 2.82^a^	60.01 ± 0.8^b^	63.06 ± 0.77^b^	75.94 ± 1.78^a^

Numbers being entered as (mean ± standard deviation). Letters that are not the same indicate a significant difference at the *P* < 0.05 level.

^a^, ^b^, ^c^, ^d^ Significant letters have been applied.

#### Drop-collapse results

The negative controls (distilled water and BHMS medium) maintained their convex and round shape after one minute, but the supernatant of the other isolates flattened to varying degrees. The results were reported as round (negative), droplets slightly tilted (+), droplets very tilted (++), and droplets completely flattened (+++) (Table 3). SHA showed the highest level of flattening of the droplet as a result of the reduction of surface tension and higher biosurfactant production (Fig. 2c).

#### Emulsion formation assay (E24)

Negative controls, consisting of MHB medium without bacteria, exhibited no emulsion formation. The isolate SHA demonstrated the highest emulsion formation rate, reaching 75% (Table 3). This outcome suggests that metabolites produced by the SHA isolate possess a greater ability to interact with hydrocarbon compounds, indicating an enhanced production of biosurfactants compared to the other isolates.

#### Results of the cell surface hydrophobicity assay (BATH)

The hydrophobicity level for all four isolates was above 58%, indicating that they are all hydrophobic, but the highest level of cell hydrophobicity was shown by the isolates SHA with 75.94% and SWOC with 73.66% (Table 3).

Consequently, these two isolates demonstrate the highest affinity for hydrocarbons, suggesting their ability to readily transition from the aqueous phase to the organic phase (oil droplets). The summary results of biosurfactant test are given in Table 3.

### Determination of crude oil removal by selected bacteria

#### Crude oil removal efficiency of selected isolates by spectrophotometry

**Fig. 3:**
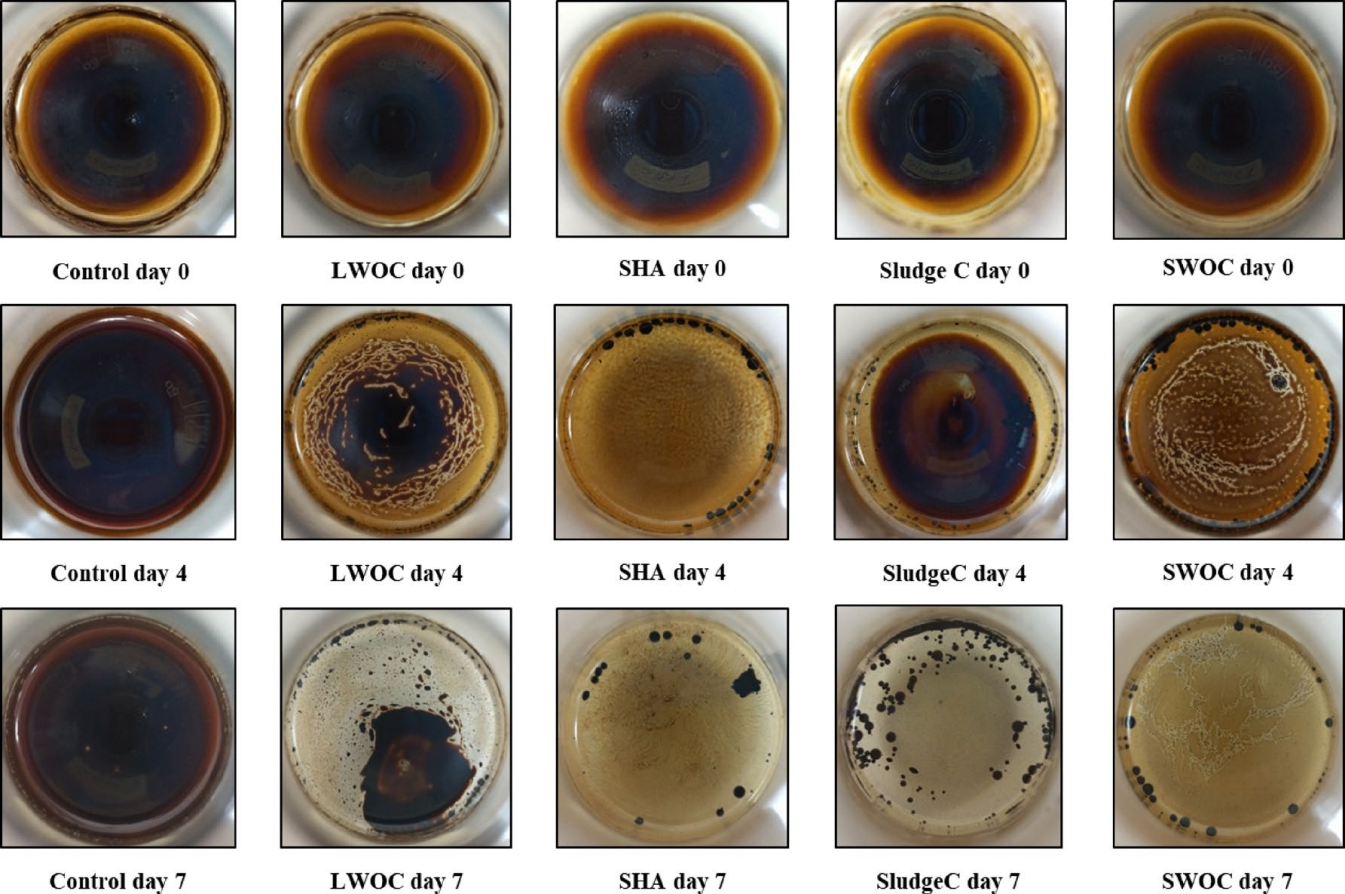
The amount of crude oil change in flasks of four selected bacteria during 7-day incubation. The flask without the presence of bacteria was used as a control.

The results of this section are shown in Fig. 3. According to the spectrophotometry measurement, the oil consumption rate for the isolates LWOE, SludgeC, SWOC, and SHA was 25%, 41%, 64%, and 71%, respectively.

#### Crude oil removal efficiency of selected isolates by GC-FID

Based on the data in Table 4, the isolates SHA and SWOC show the highest hydrocarbon degradation rates for alkanes from carbons C13 to C32. On average, the oil-removal rate of isolate SHA is 84.2% and that of isolate SWOC was 85.8%. Average degradation across short (C13–C14), medium (C15–C20), and long (C21–C32) n-alkanes was also summarized (Table 4).

**Table 4:** Percentage of n-alkane degradation in crude oil by bacteria using gas chromatography. The average degredation amount of short, medium, and long chaines of alkanes by these isolates are also reported.

Carbon number	SHA (%)	Sludge C (%)	SWOC (%)	LWOE (%)
C13	96.44	85.82	93.83	71.02
C14 (isomer 1)	68.90	65.23	83.58	46.33
C14 (isomer 2)	95.40	84.53	93.33	78.75
C15	90.94	73.35	91.13	72.16
C16	72.50	66.48	84.30	63.20
C17 (isomer 1)	81.20	67.43	86.77	65.38
C17 (isomer 2)	30.20	25.88	64.17	18.75
C18 (isomer 1)	91.39	64.84	86.69	56.81
C18 (isomer 2)	67.81	33.61	67.81	21.98
C19	88.10	56.69	85.90	59.75
C20	87.85	54.70	84.56	56.97
C21	84.58	55.82	82.53	56.10
C22	80.75	55.13	81.12	53.35
C23	85.97	61.61	84.68	53.82
C24	86.15	59.32	85.48	53.14
C25	82.02	57.25	81.78	51.23
C26	88.18	58.86	83.32	55.94
C27	75.75	58.64	81.76	53.76
C28	90.56	67.69	88.50	60.69
C29	86.20	57.93	85.21	51.91
C30	85.53	58.93	82.20	50.65
C31	90.36	62.70	89.35	38.98
C32	89.58	60.03	89.58	55.85
Average of Alkanes	**SHA**	**Sludge C**	**SWOC**	**LWOE**
C13-C14 (short chain)	86.91	78.53	90.25	65.37
C15-C20 (medium chain)	76.25	55.37	81.42	51.88
C21-C32 (long chain)	85.47	59.49	84.63	52.95

Hydrocarbon labels follow n-/iso- notation where applicable. Duplicate chain-length entries (e.g., C14, C17, C18) indicate distinct isomeric peaks detected in the crude oil. Percent reductions were calculated relative to the abiotic control processed identically

Values are reported as mean ± SD where available; results are presented for descriptive comparison among isolates

### Identification of isolates

#### Biochemical and Molecular Identification

Following spectrophotometric analysis, the two superior isolates (SWOC and SHA) were further characterized biochemically and molecularly (Table 5).

**Table 5:** Morphological characterisation, gram reaction test results and molecular identification of two superior isolates.

BACTERIAL ISOLATES ID	SHAPE OF BACTERIA	GRAM REACTION TEST	ACCESSION NUMBER	OVERLAP	SIMILARITY	SPECIES AND GENUS OF BACTERIA
SHA	Cocci	–	OQ304005	100%	99.93%	*Acinetobacter junii*
SWOC	Bacilli	+	OQ306523	100%	98.47%	*Lysinibacillus boronitolerans*

Molecular identification was performed using the 16S rRNA gene sequence. The obtained sequences were blasted against the GenBank and their homology was investigated. Similarities above 98% were reported as the genus and species of the unknown bacteria.

#### Phylogenetic tree

**Fig. 4:**
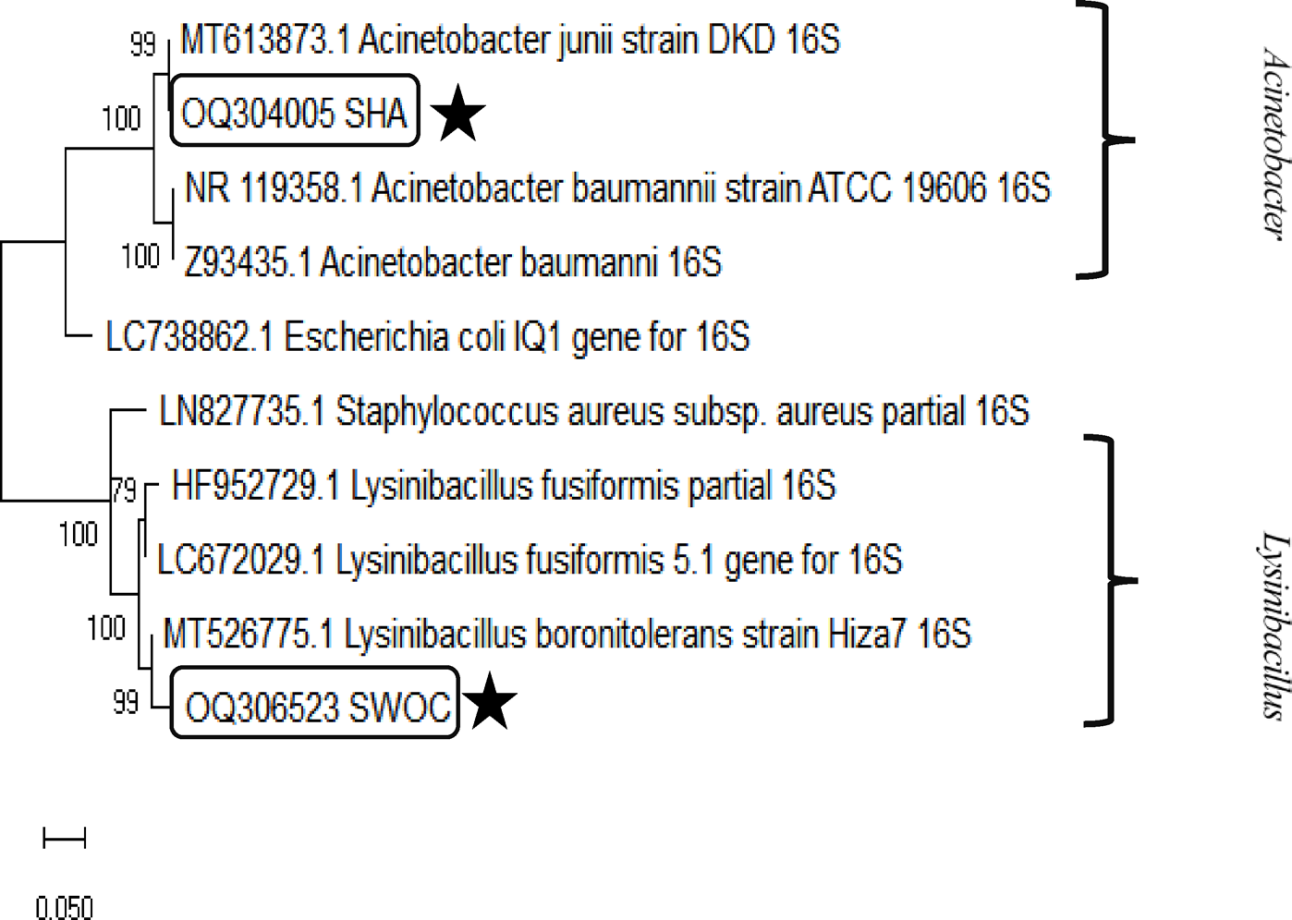
The phylogenetic tree was constructed using the Neighbor-joining method in the MEGAX software. Eight genes registered in the GenBank and two sequences (two genera) corresponding to the selected bacteria in the present study were used to draw this tree. The numbers on the lines represent bootstrap values, and the boxes around the two bacterial species indicate the samples identified in this study. Accession codes are visible next to the scientific names of the species.

By the results, the sequences obtained for species *A. junii* and *L. boronitolerans* are clustered in two separate clades among their close species of the same genera. Since these two genera are Gram-negative and Gram-positive, respectively, *Staphylococcus aureus* and *Escherichia coli* were used as outgroups for comparison, which, as expected, were placed in a separate clade from the studied species. Also, high bootstrap values from 1000 replicates strongly support the tree topology (Fig. 4).

### Results of the bacterial consortiums

The oil degradation of *Lysinibacillus boronitolerans* and *Acinetobacter junii* consortium was 55%. In contrast to the isolates cultured together, those cultured alone exhibited greater degradation efficiency. The results show that there is an antagonistic relationship between the two species.

## Discussion

Every year, large amounts of hydrocarbon pollution enter aquatic and terrestrial environments, causing irreparable damage to living organisms, including humans. The remediation of oil pollution is carried out in various ways, the most popular and affordable of which in recent years has been bioremediation. One of the most popular methods of bioremediation is the use of hydrocarbon-degrading bacteria, which use the ability of these microorganisms to remove petroleum hydrocarbons^28^. Therefore, the present study was conducted in three phases to introduce indigenous microorganisms for the remediation of oil pollution.

The primary basis for selecting bacteria was their efficiency in degrading oil in the quickest possible time. Therefore, the priority of selection was the shortest time it took for the isolates to reach the logarithmic phase in a culture medium containing oil. Because the BHMS culture medium only has hydrocarbons as a carbon source, the best oil-degrading bacteria reach the logarithmic phase more quickly^29^. Spectrophotometry also confirmed that the selected bacteria had the highest oil degradation rates after seven days.

Biosurfactants are amphiphilic compounds produced by microorganisms such as fungi, bacteria, and yeasts. These compounds have become very popular in recent decades due to their properties, such as high stability, tolerance to salinity, pH and high temperature, biodegradability, low toxicity, and environmental compatibility^2^. Biosurfactants can increase hydrocarbon solubility, mobility, and bioavailability by reducing surface and interfacial tension. Therefore, it can be stated that oil-degrading bacteria that produce more biosurfactants also have higher oil-degrading efficiency^30,31^. Five tests were used in this study to compare the superior isolates in biosurfactant production. In all tests, the isolate *Acinetobacter junii* SHA was the best-producing strain with higher efficiency than others. Especially the E24 and drop-collapse tests indicate the advantage of SHA isolate in biosurfactant production.

In the present study, five tests were used to compare the superior isolates in terms of biosurfactant production. Based on the report of Carrillo et al., the use of the blood agar hemolysis method is the most basic method for screening bacteria that produce biosurfactants. According to their report, there is a direct relationship between hemolytic activity and biosurfactant production, but this method is not specific; because sometimes the formation of a halo in this medium can be due to the production of lytic enzymes produced by some microorganisms^19,21^. Therefore, the use of other tests to confirm biosurfactant production is essential. In the oil displacement test, the displacement of the oil and the observation of a clear area on the water surface indicated the presence of biosurfactants. According to the report of Haytham et al., the larger the diameter of this halo, the more activity of the existing biosurfactant. In their research, two oil-degrading isolates formed halos with sizes of 7 cm and 6.5 cm^19,32^. In this test, all isolates formed a clear area on the water surface, with SHA having a halo diameter of 9/0 cm, the highest amount of biosurfactant produced. In the subsequent assessment, the emulsion index (E24) was employed. It should be noted that the E24 value is not always a good measure of the amount of biosurfactant produced, as some microorganisms produce high amounts of biosurfactants with low efficiency. Therefore, this index can indicate higher efficiency of the amount of biosurfactant produced^22^. In the present study, the isolate SHA had the highest E24 index. The final test for biosurfactant measurement was cell surface hydrophobicity (BATH). The ability of bacteria to adhere to hydrocarbons is one of the key abilities in oil-degrading bacteria, which allows the bacteria to have more interaction with hydrocarbons and oil degradation. In general, highly hydrophobic compounds show a degree of affinity in the range of 98–80%, while this range is 20–1% for highly hydrophilic compounds^33^. In the present study, the hydrophobicity level for all four isolates was above 58%, indicating that they are all hydrophobic, but the highest level of cell hydrophobicity was shown by the isolates SHA with 75.94% and SWOC with 73.66%.

More than 80% of crude oil is composed of hydrocarbons, of which about 60% are alkanes. Alkanes are the simplest form of petroleum hydrocarbons that can be decomposed under aerobic and anaerobic conditions^34^. For some microorganisms, alkanes with short (C3–C14) and medium (C15–C20) chains are generally easier to use than alkanes with longer carbon lengths. For other microorganisms, especially sulfate-reducing bacteria (SRB) and nitrate-reducing bacteria (NRB), shorter-chain alkanes (≤ C17) can be toxic and dissolve the bacterial membrane,for this reason, they lead to greater bacterial resistance to their decomposition. Alkanes with long chains are also often solid and have low solubility, so they are less degrade by bacteria^17^. In the present study, the percentage of hydrocarbon degradation of alkanes present in crude oil from C13 to C32 (Table 4) was calculated by comparing the gas chromatogram of the undegraded control and the decomposed sample for each isolate. The data obtained indicates that all 4 isolates were able to significantly degrade short-chain (C13 and C14), medium-chain (C20–C15), and long-chain (C21–C32) alkanes. As mentioned, long-chain alkanes are often solid and insoluble in water, so they are often not decomposed by bacteria;however, the decomposition of alkanes from C21 to C32 can be a testament to the high hydrophobicity of the bacteria. Moreover, the production of biosurfactants by bacteria leads to the formation of a disulfide bridge between the organic and aqueous phases, so that bacteria can consume hydrocarbons by transferring them to the aqueous phase^35,36^. Overall, the isolates SHA and SWOC showed the highest oil degradation rates compared to the control sample, which were 84.2% and 85.8%, respectively. These results are also consistent with the oil degradation rates determined by spectrophotometry. The results of GC analysis were consistent with the results of the oil removal measurement by spectrophotometry, and the isolates SWOC and SHA also showed the highest oil degradation rates in the spectrophotometric method.

The genus Acinetobacter is one of the most well-known oil-degrading bacteria that has been reported in various studies^7,13^. Some of the most important applications of the *Acinetobacter junii* species in recent studies include:

- Improvement of the quality of oil-contaminated soils^37^
- Increased oil degradation when used in bacterial consortium^38^
- Degradation of azo dyes with the help of bacterial enzymes^39^
- Biosynthesis of gold nanoparticles with the help of biosurfactant^40^
- High resistance to heavy metals, including lead^30,31^
- Commercial use as a producer of bioemulsifier^30,31^

In a study conducted by Zhang et al. in 2014, two *A. junii* and *Sphingomonas* sp*.* bacterial species were isolated from soil contaminated with petroleum materials in Shanghai. The results of this study showed that these two species can degrade diesel by up to 86.2% and 75.8%, respectively, within 15 days. The cell surface hydrophobicity and emulsifying activity were also reported to be 81% and 18% for *A. junii* and 94% and 24% for *Sphingomonas* sp. (Zhang et al. 2014).

Some of the important applications of different species of the genus Lysinibacillus include:

- Improvement of the quality of oil sands
- Degradation of aromatic hydrocarbons and complex hydrocarbon mixtures^41^
- Increased efficiency of diesel degradation up to 95% for hydrocarbons from C10–C28 ^41^
- Identification of a stable protease in detergents ^42^

In a study conducted by Liu et al. in 2017, more than 50 effective oil-degrading and biosurfactant-producing bacteria were isolated from soil and water contaminated with oil in the Tianjin Binhai New Area Oilfield-China, of which 24 isolates were further studied. The identified species included *Pseudomonas aeruginosa*, *Bacillus subtilis*, *Brevibacillus brevis*, *Achromobacter* sp., *Acinetobacter venetianus*, *Lysinibacillus macroides*, *Klebsiella oxytoca*, *Stenotrophomonas rhizophila*, *Rhodococcus* sp., and *Bacillus thuringiensis*. All species showed the ability to degrade more than 50% of crude oil with a concentration of 1% within seven days. Of these, eight strains were able to produce biosurfactants. In addition, environmental tolerance tests showed that most strains were able to adapt to extreme environments, including high temperatures, alkaline environments, and high salinity environments^43^.

The microbial community in nature is composed of diverse species that can have synergistic or antagonistic relationships with each other. In a bioremediation process, a particular strain may generate intermediate materials capable of serving as a substrate for another species. Consequently, some times degradation can reach completion, signifying a synergistic relationship between the involved species. On the other hand, the antagonistic relationship between species is formed when the microbial community can affect the growth and activity of other microorganisms by producing substances that have inhibitory or toxic effects. Sometimes this antagonistic relationship is also formed due to competition for a limiting substance that prevents the growth of those species^44^.

In a study conducted in 2018 by Liang et al., the effect of mixed culture on the biodegradation of crude oil was studied. In this study, four strains of CT5 (*Donghicola* sp.), CT6 (*Bacillus* sp.), CT10 (*Donghicola* sp.), and ZS1 (*Pseudomonas* sp.) were isolated from the sludge of a terminal in Zhejiang Province, China. Simultaneous growth analysis using mT-RFLP and plate inhibition methods showed that ZS1 had an antagonistic effect against CT5, CT6, and CT10. To investigate the potential compounds responsible for antagonism, the supernatant of CT10 culture was subjected to GC–MS analysis. The analysis showed that CT10 produced a number of antimicrobial compounds, including the cyclodipeptide c-(L-Pro-L-Phe), which inhibited the growth of *Pseudomonas* sp. Growth testing using c-(L-Pro-L-Phe) purified from CT10 confirmed its inhibitory activity. They also reported reduced biodegradation of crude oil in the mixed microbial culture of CT10 and ZS1^45^.

In another study conducted by Piakong et al. in 2018, the oil-degrading potential of a single species was compared to a microbial community. Three species of *Pseudomonas aeruginosa*, *Sphingomonas Paucimobilis*, and *Stenotrophomonas maltophilia* were used to investigate the oil degradation of microorganisms as single species and mixed culture. Based on observations, *P. aeruginosa* had the highest capacity to degrade crude oil compared to the microbial consortium. They attributed the inefficiency of the microbial consortium to nutrient stress and competition between the three strains^46^.

In the present study, it was also observed that in the mixed culture of the two strains *A. junii* and *L. boronitolerans,* the oil degradation rate decreases. By looking at the carbon range determination table (Table 5), it seems that this is due to competition between the limited carbon source, because these two strains have behaved similarly in most cases in the degradation of long-chain, medium-chain, and short-chain hydrocarbons, and this can be evidence of a common food source between the two species.

Antagonism observed in co-culture likely reflects a combination of resource competition (e.g., carbon or micronutrients) and potential interference mechanisms (e.g., inhibitory metabolites). Although our data are consistent with competition for carbon, the present study did not directly test inhibitory-compound production. Targeted assays such as cell-free supernatant activity, agar well-diffusion, and metabolite profiling (LC–MS/GC–MS) could help disentangle these mechanisms.

## Conclusion

Results showed that the bacteria *Lysinibacillus boronitolerans* and *Acinetobacter junii* isolated from oil-contaminated soils from Bandar Abbas and Shiraz, Iran, respectively, have high efficiency in biodegradation of crude oil within one week. However, these bacteria cannot be used as a consortium, as they have antagonistic relationship. This study also underscores the considerable potential of oil-degrading bacteria, specifically Lysinibacillus boronitolerans and Acinetobacter junii, for biosurfactant production in environmental remediation. Further investigations into bio-surfactant extractions and their industrial utilization present promising prospects for continued research endeavors.

## Data Availability

The datasets generated and/or analyzed during the current study are available from the corresponding author on reasonable request.
